# 2-(4-Fluoro­phen­yl)-5,6-methyl­enedi­oxy-3-methyl­sulfinyl-1-benzofuran

**DOI:** 10.1107/S1600536810004745

**Published:** 2010-02-13

**Authors:** Hong Dae Choi, Pil Ja Seo, Byeng Wha Son, Uk Lee

**Affiliations:** a Department of Chemistry, Dongeui University, San 24 Kaya-dong Busanjin-gu, Busan 614-714, Republic of Korea; b Department of Chemistry, Pukyong National University, 599-1 Daeyeon 3-dong, Nam-gu, Busan 608-737, Republic of Korea

## Abstract

In the title compound, C_16_H_11_FO_4_S, the O atom and the methyl group of the methyl­sulfinyl substituent are located on opposite sides of the mean plane through the 5,6-(methyl­enedi­oxy)benzofuran fragment. The 4-fluoro­phenyl ring is rotated out of the 5,6-(methyl­enedi­oxy)benzofuran plane, making a dihedral angle of 29.90 (6)°. In the crystal structure, both inter­molecular C—H⋯O hydrogen bonds link the mol­ecules into centrosymmetric dimers. The combination of C—H⋯O hydrogen bonds result in chains running along [1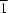 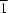].

## Related literature

For the structures of similar 5,6-methyl­enedi­oxy-1-benzofuran derivatives, see: Choi *et al.* (2007 ▶, 2009 ▶). For the pharmacological properties of benzofuran compounds, see: Howlett *et al.* (1999 ▶); Twyman & Allsop (1999 ▶). For natural products with benzofuran rings, see: Akgul & Anil (2003 ▶); von Reuss & König (2004 ▶).
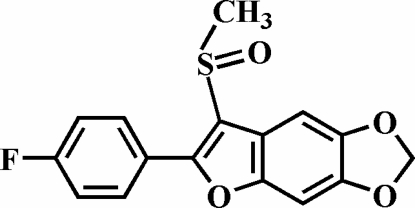


## Experimental

### 

#### Crystal data

C_16_H_11_FO_4_S
*M* *_r_* = 318.31
Triclinic, 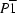

*a* = 8.0283 (7) Å
*b* = 8.4072 (7) Å
*c* = 10.6611 (9) Å
α = 85.735 (1)°
β = 86.319 (1)°
γ = 68.110 (1)°
*V* = 665.33 (10) Å^3^
*Z* = 2
Mo *K*α radiation
μ = 0.27 mm^−1^
*T* = 173 K
0.40 × 0.40 × 0.20 mm

#### Data collection

Bruker SMART APEXII CCD diffractometer
Absorption correction: multi-scan (*SADABS*; Bruker, 2009 ▶) *T*_min_ = 0.899, *T*_max_ = 0.948
4954 measured reflections
2325 independent reflections
2153 reflections with *I* > 2σ(*I*)
*R*_int_ = 0.036

#### Refinement

*R*[*F* ^2^ > 2σ(*F* ^2^)] = 0.033
*wR*(*F* ^2^) = 0.096
*S* = 1.04
2325 reflections
200 parameters
H-atom parameters constrained
Δρ_max_ = 0.76 e Å^−3^
Δρ_min_ = −0.34 e Å^−3^

### 

Data collection: *APEX2* (Bruker, 2009 ▶); cell refinement: *SAINT* (Bruker, 2009 ▶); data reduction: *SAINT*; program(s) used to solve structure: *SHELXS97* (Sheldrick, 2008 ▶); program(s) used to refine structure: *SHELXL97* (Sheldrick, 2008 ▶); molecular graphics: *ORTEP-3* (Farrugia, 1997 ▶) and *DIAMOND* (Brandenburg, 1998 ▶); software used to prepare material for publication: *SHELXL97*.

## Supplementary Material

Crystal structure: contains datablocks I. DOI: 10.1107/S1600536810004745/kj2140sup1.cif

Structure factors: contains datablocks I. DOI: 10.1107/S1600536810004745/kj2140Isup2.hkl

Additional supplementary materials:  crystallographic information; 3D view; checkCIF report

## Figures and Tables

**Table 1:** Hydrogen-bond geometry (Å, °)

*D*—H⋯*A*	*D*—H	H⋯*A*	*D*⋯*A*	*D*—H⋯*A*
C3—H3⋯O2^i^	0.93	2.54	3.383 (2)	151
C14—H14⋯O4^ii^	0.93	2.57	3.368 (2)	144

Symmetry codes: (i) 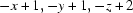; (ii) 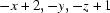.

**Fig. 1.**
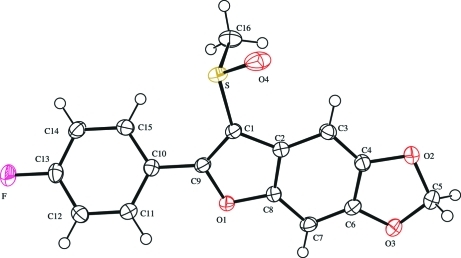
The molecular structure of the title compound with the atom numbering scheme. Displacement ellipsoids are drawn at the 50 % probability level.

**Fig. 2.**
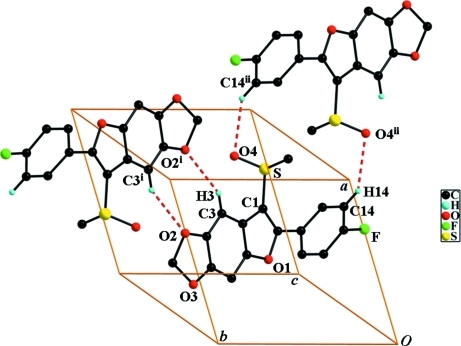
C—H···O interactions (dotted lines) in the crystal structure of the title compound. [Symmetry codes: (i) - x + 1, - y + 1, - z + 2; (ii) - x + 2, - y, - z + 1.]

## supplementary crystallographic information

### Comment

Benzofuran ring systems have received considerable attention in view of their pharmacological properties (Howlett *et al.*, 1999; Twyman & Allsop, 1999) and their occurrence as natural products (Akgul & Anil, 2003; von Reuss & König, 2004). As a part of our ongoing studies of the effect of side chain substituents on the solid state structures of 5,6-methylenedioxy-1-benzofuran analogues (Choi *et al.*, 2007,2009), we report the crystal structure of the title compound (Fig.1).

The 5,6-(methylenedioxy)benzofuran unit is essentially planar, with a mean deviation of 0.060 (2) Å from the least-squares plane defined by the twelve constituent atoms. The dihedral angle formed by the plane of the 5,6-(methylenedioxy)benzofuran ring and the plane of 4-fluorophenyl ring is 29.90 (6).

In the crystal packing, both intermolecular C—H···O hydrogen bonds (C3—H3···O2 and C14—H14···O4) link the molecules into dimers (Table 1, Figure 2). Together, the C—H···O hydrogen-bond interactions link the molecules into a one-dimensional chain running in the [1,-1,-1] direction.

### Experimental

77% 3-chloroperoxybenzoic acid (247 mg, 1.1 mmol) was added in small portions to a stirred solution of 2-(4-fluorophenyl)-5,6-methylenedioxy-3-methylsulfanyl-1-benzofuran (302 mg, 1.0 mmol) in dichloromethane (30 ml) at 273 K. After being stirred at room temperature for 2h, the mixture was washed with saturated sodium bicarbonate solution and the organic layer was separated, dried over magnesium sulfate, filtered and concentrated in vacuum. The residue was purified by column chromatography (hexane–ethyl acetate, 1:1 v/v) to afford the title compound as a colorless solid [yield 71%, m.p. 488–489 K; *R*_f_ = 0.51 (hexane–ethyl acetate, 1:2 v/v)]. Single crystals suitable for X-ray diffraction were prepared by slow evaporation of a solution of the title compound in chloroform at room temperature.

### Refinement

All H atoms were geometrically positioned and refined using a riding model, with C—H = 0.93 Å for aryl, 0.97 Å for methylene, and 0.96 Å for methyl H atoms. *U*_iso_(H) = 1.2*U*_eq_(C) for aryl and methylene H atoms, and 1.5*U*_eq_(C) for methyl H atoms.

### Crystal data

**Table:**

C_16_H_11_FO_4_S	*Z* = 2
*M**_r_* = 318.31	*F*(000) = 328
Triclinic, *P*1	*D*_x_ = 1.589 Mg m^−^^3^
Hall symbol: -P 1	Mo *K*α radiation, λ = 0.71073 Å
*a* = 8.0283 (7) Å	Cell parameters from 4336 reflections
*b* = 8.4072 (7) Å	θ = 2.6–27.4°
*c* = 10.6611 (9) Å	µ = 0.27 mm^−^^1^
α = 85.735 (1)°	*T* = 173 K
β = 86.319 (1)°	Block, colourless
γ = 68.110 (1)°	0.40 × 0.40 × 0.20 mm
*V* = 665.33 (10) Å^3^	

### Data collection

**Table:**

Bruker SMART APEXII CCD diffractometer	2325 independent reflections
Radiation source: Rotating Anode	2153 reflections with *I* > 2σ(*I*)
HELIOS	*R*_int_ = 0.036
Detector resolution: 10.0 pixels mm^-1^	θ_max_ = 25.0°, θ_min_ = 1.9°
φ and ω scans	*h* = −9→9
Absorption correction: multi-scan (*SADABS*; Bruker, 2009)	*k* = −9→9
*T*_min_ = 0.899, *T*_max_ = 0.948	*l* = −12→12
4954 measured reflections	

### Refinement

**Table:**

Refinement on *F*^2^	Primary atom site location: structure-invariant direct methods
Least-squares matrix: full	Secondary atom site location: difference Fourier map
*R*[*F*^2^ > 2σ(*F*^2^)] = 0.033	Hydrogen site location: difference Fourier map
*wR*(*F*^2^) = 0.096	H-atom parameters constrained
*S* = 1.04	*w* = 1/[σ^2^(*F*_o_^2^) + (0.0477*P*)^2^ + 0.4337*P*] where *P* = (*F*_o_^2^ + 2*F*_c_^2^)/3
2325 reflections	(Δ/σ)_max_ < 0.001
200 parameters	Δρ_max_ = 0.76 e Å^−^^3^
0 restraints	Δρ_min_ = −0.34 e Å^−^^3^

### Special details

**Table:**

Geometry. All esds (except the esd in the dihedral angle between two l.s. planes) are estimated using the full covariance matrix. The cell esds are taken into account individually in the estimation of esds in distances, angles and torsion angles; correlations between esds in cell parameters are only used when they are defined by crystal symmetry. An approximate (isotropic) treatment of cell esds is used for estimating esds involving l.s. planes.
Refinement. Refinement of F^2^ against ALL reflections. The weighted R-factor wR and goodness of fit S are based on F^2^, conventional R-factors R are based on F, with F set to zero for negative F^2^. The threshold expression of F^2^ > 2sigma(F^2^) is used only for calculating R-factors(gt) etc. and is not relevant to the choice of reflections for refinement. R-factors based on F^2^ are statistically about twice as large as those based on F, and R- factors based on ALL data will be even larger.

### Fractional atomic coordinates and isotropic or equivalent isotropic displacement parameters (Å^2^)

**Table:**

	*x*	*y*	*z*	*U*_iso_*/*U*_eq_	
S	0.80463 (5)	0.18763 (6)	0.60869 (4)	0.02335 (16)	
F	0.68941 (16)	−0.01363 (16)	0.02490 (11)	0.0385 (3)	
O1	0.29585 (16)	0.35961 (16)	0.51174 (11)	0.0228 (3)	
O2	0.25335 (17)	0.57506 (17)	0.98364 (12)	0.0292 (3)	
O3	−0.01602 (17)	0.65674 (17)	0.88470 (12)	0.0285 (3)	
O4	0.84924 (18)	0.30301 (18)	0.68884 (15)	0.0372 (4)	
C1	0.5684 (2)	0.2707 (2)	0.59585 (16)	0.0206 (4)	
C2	0.4380 (2)	0.3671 (2)	0.68856 (16)	0.0204 (4)	
C3	0.4467 (2)	0.4134 (2)	0.81182 (16)	0.0225 (4)	
H3	0.5537	0.3792	0.8537	0.027*	
C4	0.2853 (2)	0.5124 (2)	0.86443 (16)	0.0229 (4)	
C5	0.0711 (3)	0.6920 (2)	0.98677 (18)	0.0289 (4)	
H5A	0.0661	0.8093	0.9776	0.035*	
H5B	0.0117	0.6769	1.0664	0.035*	
C6	0.1218 (2)	0.5638 (2)	0.80438 (17)	0.0222 (4)	
C7	0.1075 (2)	0.5200 (2)	0.68512 (17)	0.0237 (4)	
H7	−0.0006	0.5531	0.6449	0.028*	
C8	0.2736 (2)	0.4202 (2)	0.63084 (16)	0.0209 (4)	
C9	0.4775 (2)	0.2695 (2)	0.49235 (16)	0.0214 (4)	
C10	0.5313 (2)	0.1926 (2)	0.37027 (16)	0.0214 (4)	
C11	0.4330 (2)	0.2717 (2)	0.26424 (16)	0.0227 (4)	
H11	0.3323	0.3721	0.2719	0.027*	
C12	0.4845 (2)	0.2015 (2)	0.14773 (17)	0.0251 (4)	
H12	0.4192	0.2530	0.0769	0.030*	
C13	0.6347 (2)	0.0537 (2)	0.13962 (17)	0.0259 (4)	
C14	0.7337 (2)	−0.0306 (2)	0.24219 (17)	0.0272 (4)	
H14	0.8334	−0.1315	0.2336	0.033*	
C15	0.6797 (2)	0.0397 (2)	0.35841 (17)	0.0258 (4)	
H15	0.7430	−0.0156	0.4292	0.031*	
C16	0.8251 (3)	−0.0009 (3)	0.70708 (19)	0.0321 (4)	
H16A	0.9492	−0.0629	0.7248	0.048*	
H16B	0.7803	−0.0726	0.6646	0.048*	
H16C	0.7568	0.0320	0.7845	0.048*	

### Atomic displacement parameters (Å^2^)

**Table:**

	*U*^11^	*U*^22^	*U*^33^	*U*^12^	*U*^13^	*U*^23^
S	0.0165 (2)	0.0224 (3)	0.0292 (3)	−0.00522 (18)	−0.00198 (17)	0.00089 (18)
F	0.0361 (7)	0.0477 (7)	0.0256 (6)	−0.0067 (6)	0.0027 (5)	−0.0155 (5)
O1	0.0185 (6)	0.0260 (7)	0.0216 (6)	−0.0047 (5)	−0.0023 (5)	−0.0047 (5)
O2	0.0273 (7)	0.0341 (7)	0.0212 (6)	−0.0043 (6)	−0.0010 (5)	−0.0076 (5)
O3	0.0223 (7)	0.0311 (7)	0.0269 (7)	−0.0034 (5)	0.0030 (5)	−0.0071 (5)
O4	0.0264 (7)	0.0293 (8)	0.0570 (10)	−0.0088 (6)	−0.0137 (6)	−0.0068 (7)
C1	0.0175 (8)	0.0201 (8)	0.0224 (9)	−0.0050 (7)	−0.0004 (7)	−0.0011 (7)
C2	0.0193 (8)	0.0197 (8)	0.0217 (8)	−0.0065 (7)	−0.0012 (7)	−0.0004 (7)
C3	0.0210 (9)	0.0248 (9)	0.0218 (9)	−0.0082 (7)	−0.0034 (7)	−0.0004 (7)
C4	0.0262 (9)	0.0227 (9)	0.0194 (8)	−0.0083 (7)	−0.0011 (7)	−0.0018 (7)
C5	0.0283 (10)	0.0282 (10)	0.0266 (9)	−0.0056 (8)	0.0026 (8)	−0.0080 (8)
C6	0.0196 (9)	0.0194 (9)	0.0255 (9)	−0.0052 (7)	0.0020 (7)	−0.0009 (7)
C7	0.0175 (8)	0.0243 (9)	0.0271 (9)	−0.0050 (7)	−0.0030 (7)	−0.0013 (7)
C8	0.0217 (9)	0.0210 (9)	0.0197 (8)	−0.0072 (7)	−0.0015 (7)	−0.0026 (7)
C9	0.0176 (8)	0.0207 (9)	0.0236 (9)	−0.0044 (7)	−0.0003 (7)	−0.0010 (7)
C10	0.0212 (9)	0.0222 (9)	0.0219 (9)	−0.0093 (7)	0.0003 (7)	−0.0031 (7)
C11	0.0204 (9)	0.0211 (9)	0.0259 (9)	−0.0069 (7)	−0.0010 (7)	−0.0018 (7)
C12	0.0261 (9)	0.0283 (10)	0.0219 (9)	−0.0109 (8)	−0.0034 (7)	0.0000 (7)
C13	0.0274 (10)	0.0310 (10)	0.0218 (9)	−0.0130 (8)	0.0028 (7)	−0.0089 (7)
C14	0.0228 (9)	0.0252 (10)	0.0303 (10)	−0.0038 (7)	−0.0005 (7)	−0.0076 (8)
C15	0.0249 (9)	0.0254 (9)	0.0249 (9)	−0.0061 (8)	−0.0051 (7)	−0.0009 (7)
C16	0.0292 (10)	0.0277 (10)	0.0383 (11)	−0.0100 (8)	−0.0077 (8)	0.0079 (8)

### Geometric parameters (Å, °)

**Table:**

S—O4	1.4903 (14)	C5—H5B	0.9700
S—C1	1.7707 (17)	C6—C7	1.373 (3)
S—C16	1.7944 (19)	C7—C8	1.398 (2)
F—C13	1.363 (2)	C7—H7	0.9300
O1—C8	1.380 (2)	C9—C10	1.464 (2)
O1—C9	1.380 (2)	C10—C15	1.397 (3)
O2—C4	1.384 (2)	C10—C11	1.398 (2)
O2—C5	1.426 (2)	C11—C12	1.387 (3)
O3—C6	1.379 (2)	C11—H11	0.9300
O3—C5	1.436 (2)	C12—C13	1.375 (3)
C1—C9	1.364 (2)	C12—H12	0.9300
C1—C2	1.443 (2)	C13—C14	1.380 (3)
C2—C8	1.395 (2)	C14—C15	1.386 (3)
C2—C3	1.411 (2)	C14—H14	0.9300
C3—C4	1.363 (3)	C15—H15	0.9300
C3—H3	0.9300	C16—H16A	0.9600
C4—C6	1.401 (3)	C16—H16B	0.9600
C5—H5A	0.9700	C16—H16C	0.9600
			
O4—S—C1	107.29 (8)	O1—C8—C2	110.69 (15)
O4—S—C16	105.76 (9)	O1—C8—C7	123.97 (15)
C1—S—C16	98.28 (9)	C2—C8—C7	125.34 (16)
C8—O1—C9	106.48 (13)	C1—C9—O1	110.48 (15)
C4—O2—C5	105.53 (13)	C1—C9—C10	133.97 (16)
C6—O3—C5	105.04 (14)	O1—C9—C10	115.53 (14)
C9—C1—C2	107.49 (15)	C15—C10—C11	119.35 (16)
C9—C1—S	126.25 (13)	C15—C10—C9	120.68 (16)
C2—C1—S	126.00 (13)	C11—C10—C9	119.97 (16)
C8—C2—C3	120.39 (16)	C12—C11—C10	120.44 (16)
C8—C2—C1	104.84 (15)	C12—C11—H11	119.8
C3—C2—C1	134.76 (16)	C10—C11—H11	119.8
C4—C3—C2	114.39 (16)	C13—C12—C11	118.26 (16)
C4—C3—H3	122.8	C13—C12—H12	120.9
C2—C3—H3	122.8	C11—C12—H12	120.9
C3—C4—O2	126.99 (16)	F—C13—C12	118.70 (16)
C3—C4—C6	124.06 (16)	F—C13—C14	118.04 (16)
O2—C4—C6	108.93 (15)	C12—C13—C14	123.25 (17)
O2—C5—O3	107.68 (14)	C13—C14—C15	117.97 (17)
O2—C5—H5A	110.2	C13—C14—H14	121.0
O3—C5—H5A	110.2	C15—C14—H14	121.0
O2—C5—H5B	110.2	C14—C15—C10	120.68 (17)
O3—C5—H5B	110.2	C14—C15—H15	119.7
H5A—C5—H5B	108.5	C10—C15—H15	119.7
C7—C6—O3	127.18 (16)	S—C16—H16A	109.5
C7—C6—C4	123.29 (16)	S—C16—H16B	109.5
O3—C6—C4	109.50 (15)	H16A—C16—H16B	109.5
C6—C7—C8	112.52 (16)	S—C16—H16C	109.5
C6—C7—H7	123.7	H16A—C16—H16C	109.5
C8—C7—H7	123.7	H16B—C16—H16C	109.5
			
O4—S—C1—C9	144.09 (16)	C3—C2—C8—O1	−179.45 (15)
C16—S—C1—C9	−106.47 (17)	C1—C2—C8—O1	1.26 (19)
O4—S—C1—C2	−29.26 (18)	C3—C2—C8—C7	0.1 (3)
C16—S—C1—C2	80.17 (17)	C1—C2—C8—C7	−179.22 (17)
C9—C1—C2—C8	−0.86 (19)	C6—C7—C8—O1	179.98 (16)
S—C1—C2—C8	173.53 (13)	C6—C7—C8—C2	0.5 (3)
C9—C1—C2—C3	−179.99 (19)	C2—C1—C9—O1	0.2 (2)
S—C1—C2—C3	−5.6 (3)	S—C1—C9—O1	−174.20 (12)
C8—C2—C3—C4	−0.8 (2)	C2—C1—C9—C10	−178.74 (18)
C1—C2—C3—C4	178.23 (18)	S—C1—C9—C10	6.9 (3)
C2—C3—C4—O2	179.41 (16)	C8—O1—C9—C1	0.61 (19)
C2—C3—C4—C6	1.0 (3)	C8—O1—C9—C10	179.74 (14)
C5—O2—C4—C3	171.11 (18)	C1—C9—C10—C15	30.2 (3)
C5—O2—C4—C6	−10.26 (19)	O1—C9—C10—C15	−148.66 (16)
C4—O2—C5—O3	17.49 (19)	C1—C9—C10—C11	−150.3 (2)
C6—O3—C5—O2	−18.03 (19)	O1—C9—C10—C11	30.9 (2)
C5—O3—C6—C7	−170.31 (18)	C15—C10—C11—C12	−1.5 (3)
C5—O3—C6—C4	11.72 (19)	C9—C10—C11—C12	178.93 (16)
C3—C4—C6—C7	−0.4 (3)	C10—C11—C12—C13	−0.5 (3)
O2—C4—C6—C7	−179.07 (16)	C11—C12—C13—F	−178.17 (16)
C3—C4—C6—O3	177.68 (16)	C11—C12—C13—C14	1.8 (3)
O2—C4—C6—O3	−1.0 (2)	F—C13—C14—C15	178.87 (17)
O3—C6—C7—C8	−178.09 (16)	C12—C13—C14—C15	−1.1 (3)
C4—C6—C7—C8	−0.4 (3)	C13—C14—C15—C10	−1.0 (3)
C9—O1—C8—C2	−1.19 (19)	C11—C10—C15—C14	2.3 (3)
C9—O1—C8—C7	179.28 (16)	C9—C10—C15—C14	−178.20 (17)

### Hydrogen-bond geometry (Å, °)

**Table:**

*D*—H···*A*	*D*—H	H···*A*	*D*···*A*	*D*—H···*A*
C3—H3···O2^i^	0.93	2.54	3.383 (2)	151
C14—H14···O4^ii^	0.93	2.57	3.368 (2)	144

Symmetry codes: (i) −*x*+1, −*y*+1, −*z*+2; (ii) −*x*+2, −*y*, −*z*+1.
